# Metastatic malignant melanoma of the conjunctiva: a case report

**DOI:** 10.1186/1757-1626-2-125

**Published:** 2009-02-04

**Authors:** Nikolaos Manidakis, Ioannis Polyzois, Euaggelos Tsialogiannis, Maria Marples, Andrew Boon, Eleftherios Tsiridis

**Affiliations:** 1 Academic Unit of Trauma & Orthopaedic Surgery, Leeds General Infirmary, Leeds School of Medicine, Leeds, LS1 3EX, UK; 2 Department of Clinical Oncology, St James University Hospital, Beckett Street, Leeds, LS9 7TF, UK; 3 Department of Histopathology, St James University Hospital, Beckett Street, Leeds, LS9 7TF, UK

## Abstract

**Background:**

Malignant melanoma of the conjunctiva is an extremely rare non-cutaneous neoplasm with infrequent skeletal metastatic spread.

**Case presentation:**

We present the case of a 54 year old female Caucasian patient with osseous metastases originating from a malignant melanoma of her right conjunctiva. Metastatic deposits were identified in the left humeral diaphysis and left tibial metaphysis. Clinical, radiological and scintigraphic evaluation necessitated prompt stabilisation of both long bones. Following reamed intramedullary nailing and post-operative radiotherapy she remains asymptomatic six months post-operatively.

**Conclusion:**

This unusual pattern of metastatic spread to the appendicular skeleton of an extremely rare melanomatous lesion requires diagnostic vigilance as well as a multidisciplinary approach for accurate diagnosis, staging and management. Due to the poor prognosis, treatment goals should be directed to palliation of symptoms and prolongation of the quality of life.

## Introduction

Malignant melanoma of the conjunctiva is a rare extraocular neoplasm. Its incidence is reported to be 0.2–0.8 per million in white populations and represents 1.6% of all non-cutaneous melanomas [1].

Although the potential metastastic spread of conjunctival melanoma to bone has been recognized [2], the available literature does not accurately specify the exact skeletal sites and number of patients affected. Most of the information about melonomatous spread to bone derives from studies concentrated on the cutaneous type with a reported incidence of 7 – 17% [3-5], tendency for spread to the axial skeleton [3,4] and a mean survival of 3.6 – 8 months [3,4,6] despite treatment.

A case of metastatic spread of malignant conjunctival melanoma to the appendicular skeleton is presented.

## Case report

A 54 year old female Caucasian patient presented to the orthopaedic outpatient clinic complaining of left arm and left knee pain. She had been diagnosed with malignant melanoma of the palpebral and forniceal conjunctiva 2.5 years before and had received treatment at the regional ocular oncology centre in the form of surgical excision, cryotherapy and adjuvant Mitomycin C.

During her oncological follow up 30 months following the original diagnosis, multiple bilateral pulmonary metastases all measuring <0.5 cms in diameter as well as a 4 × 2 cm right ischial lesion were identified. She was complaining of left arm and left knee pain and was therefore referred to the orthopaedic clinic.

The pattern of the pain was constant with occasional nocturnal exacerbations and was aggravated by weight bearing activities of both extremities. Clinical examination demonstrated localized areas of tenderness over the midshaft area of the left humerus as well as over the anteromedial aspect of the left proximal tibia. Furthermore a non-fluctuant swelling measuring 2 × 2 centimeters in diameter was palpable over the corresponding left proximal tibial metaphysis. All neighbouring joints examined normal. Her right ischial lesion was asymptomatic.

**Figure 1:**
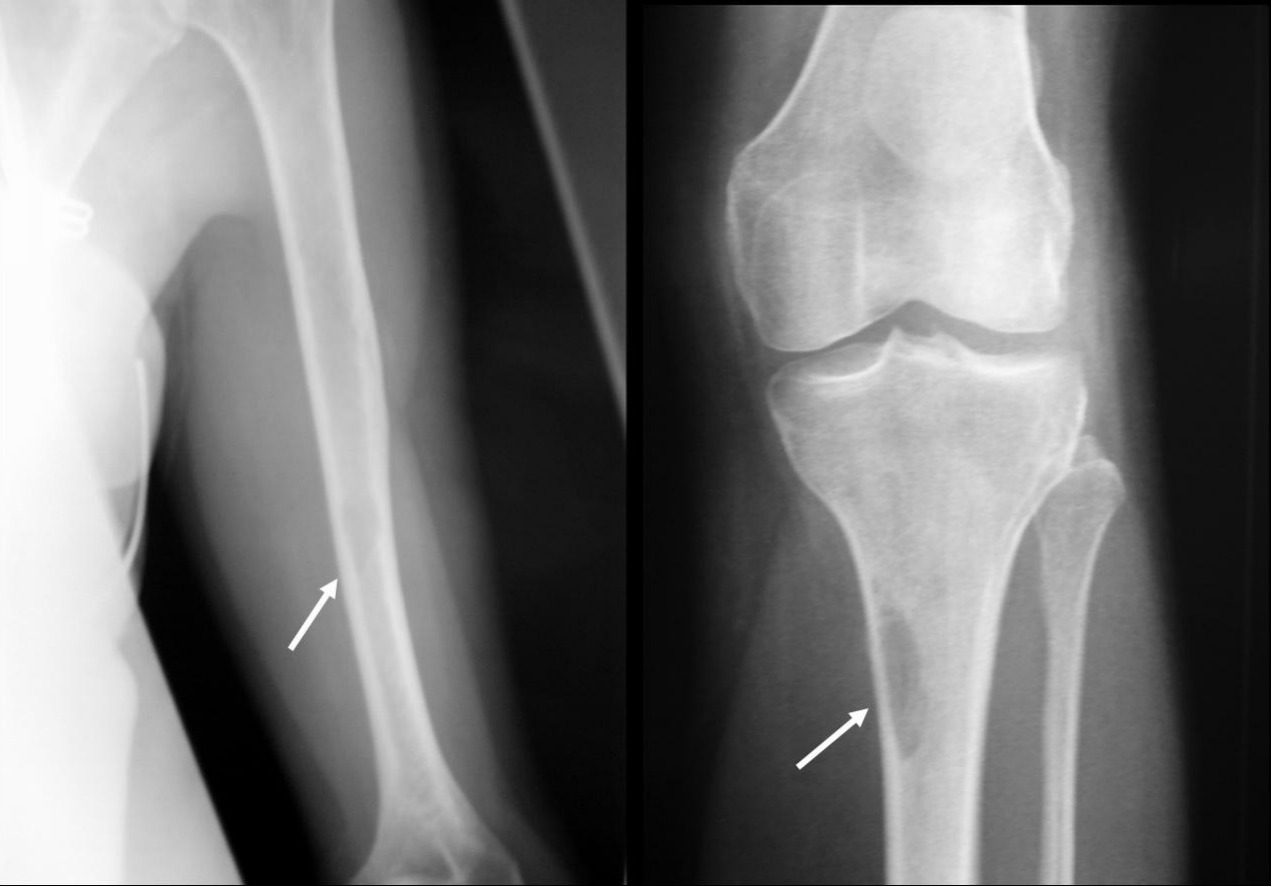
**Pre-operative radiographs of the left humerus and left knee demonstrating osteolytic lesions eroding the cortex**.

Radiographic evaluation demonstrated lytic lesions of the left humeral diaphysis and proximal tibial metaphysis with associated cortical involvement and ill defined zones of transition, however no evidence of fracture (Figure 1). A bone scan confirmed the presence of three active areas corresponding to the lucent radiographic areas of the left humerus, left tibia and right ischium. It did not identify any further skeletal lesions

Regarding her osseous lesions she underwent intramedullary nailing procedures of both long bones on a semi-elective basis due to the danger of impending fracture. According to the Mirels classification for metastatic bone disease at risk of fracture, [7] she scored 9 points for the humerus and 10 for the tibia.

**Figure 2:**
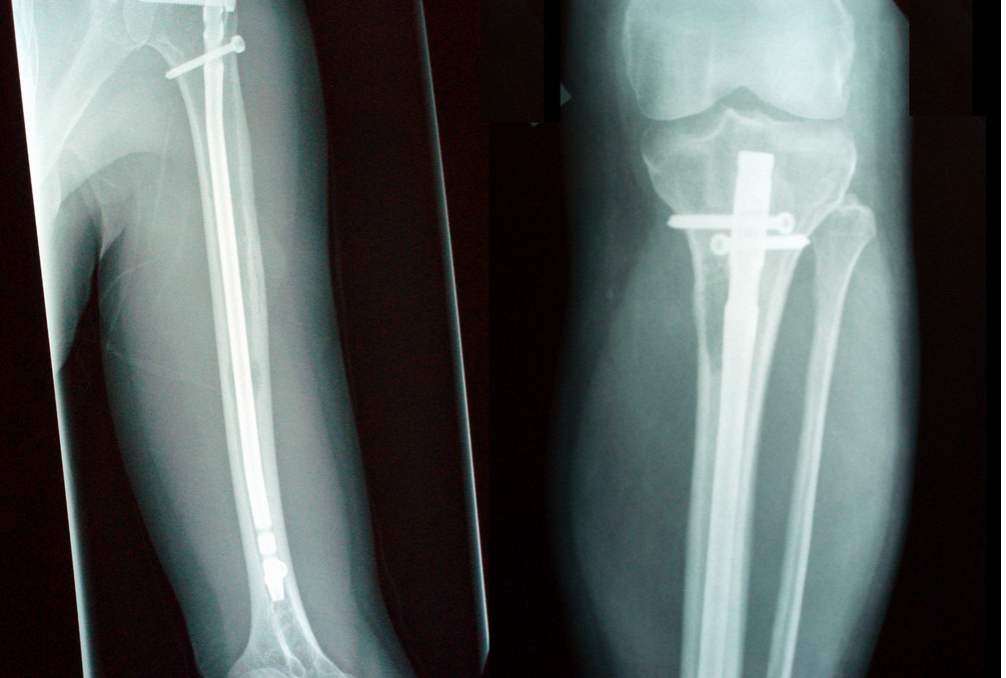
**Post-operative radiographs demonstrating stabilized left humerus and left tibia with intramedullary nails**.

A standard reamed and locked nailing technique was applied for stabilization of both bones with no associated intra-operative complications (Figure 2). Venting of both bones was performed during the procedure in an attempt to reduce intra-osseous pressure and prevent the risk of fat and tumour embolisation. Intramedullary reamings, as well as specimens obtained via open curettage of the radiologically suspect lesions were sent to the histopathology department.

**Figure 3:**
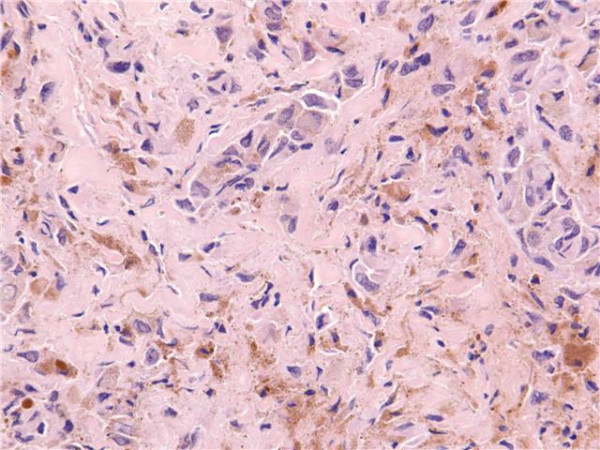
**Histological section of biopsy**. Original magnification ×200, H&E stain showing strands of focally pigmented pleomorphic epithelioid cells in decalcified sections of bone. Features are fully consistent with metastatic melanoma.

The patient had an uneventful recovery and was discharged home within 4 days. Histological examination confirmed the presence of devitalized bone and extensively necrotic, heavily pigmented epithelioid tumour, consistent with a diagnosis of metastatic malignant melanoma. There was a low mitotic count (1/mm^2^) and no significant lymphocytic infiltrate (Figure 3).

At her latest follow up visit in the orthopaedic clinic 6 months post-operatively, she remains pain-free with no radiographic evidence of further metastatic spread of the involved bones. Further oncological input consisted of palliative radiotherapy to her right ischial and left humeral lesions, as well as bisphosphonate administration. No conjunctival recurrence has been identified 3 years following the original diagnosis.

## Discussion

Malignant melanoma of the conjunctiva is a potentially lethal neoplasm with an average 10 year mortality rate of 30% [2]. Tumours located in the palpebral and forniceal conjunctival regions are infrequent (incidence of 4% and 3% respectively) and are associated with a worse prognosis [1], however the extent of the disease at time of presentation is the most important factor in determining outcome. The conjunctival stroma contains blood vessels and lymphatics thus local and systemic metastases can occur [2]. Regional lymph node involvement usually occurs first, however systemic dissemination without previous lymphatic spread can take place [2].

Osseous metastases occur in patients with advanced primary and multiple lesions. They generally exhibit a predilection to spread in the axial skeleton with an incidence of 70–86% [3,4]. This can be partly explained by red marrow predominance in the axial skeleton and a unique vascular venous network anastomosing the pelvis to the spine, resulting in an increased likelihood of seeding [6]. Metastases to the appendicular skeleton occur infrequently and when isolated they are associated with a three fold increased survival [6]. The melanocytes produce factors that stimulate osteoclasts, stromal cells and macrophages to release osteotropic cytokines such as Interleukin-6 (IL-6), Prostaglandin E2 (PGE2) and Transforming Growth Factor alpha (TGF-α) [8]. Osseous metastases are generally osteolytic, eccentric and oval, provoking little if any periosteal response [3,4]. They first involve the medullary cavity or trabeculae and proceed to cortical erosion, leading to pathological fractures, bone marrow failure and neurological deficits.

In trying to establish the presence of metastatic spread of conjunctival melanoma for staging purposes, several methods are available. Magnetic Resonance Imaging (MRI) of the head is a useful tool in assessing for orbital or other regional spread. The latter can also be detected by sentinel lymph node excision biopsy which offers the potential for both earlier diagnosis and provision of adjuvant therapies [2].

Osseous metastases can be identified by several imaging methods. The features of melanomatous lesions on plain radiographs have already been discussed. Bone scintigraphy is more sensitive in being able to detect more lesions and earlier [3]. Although computed tomography (CT) can identify skeletal metastases, MRI can also assess soft tissue extent and can influence treatment, despite its cost [5]. Positron emission tomography CT can be useful for the follow up and restaging of conjunctival melanoma; however its use in initial staging is limited [9].

In the present case two symptomatic osteolytic metastatic foci were identified in the humeral diaphysis and proximal tibial metaphysis. There were signs of cortical erosion which threatened impending pathological fractures of both long bones, according to the Mirels scoring system [7]. Although previous recommendations favoured the use of palliative fixation for unstable pathological fractures [4], intramedullary nailing facilitates stabilization of the whole bone, which is mandatory for prophylaxis against impending fractures [10]. Venting during prophylactic reamed nailing, reduces pressurization of the canal, therefore this technique was used as a measure against fat and tumour embolisation [11]. The use of reamings as histological specimens is not supported in the literature, as bone sampling can be destructive, making diagnosis difficult and unreliable, therefore they should be accompanied by biopsies, as was done in this case [12].

The presence of multiple foci to the skeleton and lungs precluded metastasectomy as this would have been associated with significant morbidity [13]. Adjuvant therapeutic regimens were limited to the use of radiotherapy of the ischial and humeral lesions as well as bisphosphonate administration. Radiotherapy prevents local recurrence and provides palliation, despite previous concerns regarding radioresistance of malignant melanoma [14]. Furthermore it has been reported that appendicular metastases respond better. Bisphosphonates induce apoptosis in human melanoma cells, can reduce bone pain, and can inhibit osteoclast activity [15]. Systemic chemotherapy, biochemotherapy and immunotherapy can be used; however survival as well as control of lesion growth and pain is generally not improved [4]. Isolated limb perfusion chemotherapy can achieve symptomatic and radiologic control, however it was not used in this case[13]

## Conclusion

Malignant melanoma of the conjunctiva is an extremely rare neoplasm. Palbebral and forniceal involvement, although uncommon, can be associated with poorer prognosis and survival. Systemic spread to the skeleton and particularly the long bones of the extremities, without previous regional lymphatic involvement, adds to the infrequency of occurrence and undermines the prognosis of the herein presented case.

In the presence of impending pathological fractures, osseous surgical stabilization followed by adjuvant therapies should be offered in an attempt to improve the patient's quality of life. Combined input from the opthalmological, oncological, radiological, histopathological and orthopaedic disciplines is required for accurate diagnosis, staging and management.

## Consent

Written informed consent was obtained from the patient for publication of this case report and accompanying images in Journal of Medical Case Reports.

## Competing interests

The authors declare that they have no competing interests.

## Authors' contributions

NM conducted a literature search and prepared the final manuscript. IP conducted a literature search and contributed to the preparation of the manuscript. ET prepared the first draft of the manuscript. MM helped in preparing the first draft of the manuscript and treated the patient, AB prepared the histological slides and contributed to the final manuscript, ET supervised the manuscript and treated the patient. All authors contributed equally in collecting patient data and editing radiographic images.
